# Human-Centered Design and Sustainable Malaria Interventions

**DOI:** 10.9745/GHSP-D-19-00189

**Published:** 2019-06-24

**Authors:** Michael Macdonald, Thomas Putzer

**Affiliations:** a Associate Editor, Global Health: Science and Practice Journal, and Consultant, Catonsville, MD, USA.; b SC Johnson & Son, Inc., Racine, WI, USA.

## Abstract

Human-centered design provides a method to adapt malaria control interventions to be more closely aligned with a family's convenience, comfort, and personal lifestyle, enabling a broader and more sustained culture of access and use.

See related article by Kim.

*For every complex problem there is an answer that is clear, simple, and wrong.* - H.L. Mencken (paraphrased), *Prejudices: Second Series* (1920)

The article by Kim et al.,^1^ “Using a human-centered design approach to determine consumer preferences for long-lasting insecticidal nets in Ghana,” raises 2 important questions. First, can the public sector adapt long-lasting insecticidal net (LLIN) designs to the user's point of view rather than purely technical and cost considerations? Second, is it possible to segment the market for mosquito nets such that people who can afford to pay can acquire the nets they want commercially, while the public sector focuses limited resources on those most in need who are unable to afford to purchase nets?

These questions challenge current strategies on 2 counts. First, large international tenders for mosquito nets are awarded on unit costs that meet the minimum physical standards. Second, “universal coverage” demands that *all* persons living in malaria endemic areas receive a free standard net.

While treated mosquito nets can have a significant impact on malaria illness and death,^2^ some estimate that, depending on the community being studied, as much as half of the households that receive nets do not use them for their intended purpose.^3^ Excessive heat and reduced airflow are often cited as barriers to LLIN use,^4^ with reduced air flow ranging from 55% to 71% in 1 study of 11 commercial nets.^5^ Imagine how much more impact LLINs could make if design changes to increase end-user compliance were addressed.

Kim et al. explored the barriers to LLIN ownership and use among middle-class Ghanaians through a human-centered design (HCD) process that moves beyond the traditional public health tools of focus group discussions, household surveys, and trials of improved practices:

*The result was a rich mix of data and the identification of key consumer insights regarding middle-class Ghanaians' perceptions of self, their behaviors and attitudes related to malaria prevention, and their use of LLINs.*

The study found:

*… in most accounts [free public-sector LLINs] were inconvenient, uncomfortable, and not aesthetically pleasing, thus they were undesirable to use.*

Suggested changes to the standard LLINs included a more convenient way to hang the net, a more attractive silhouette, and a zipper for ease of entry and exit. Previous LLIN design work in Ghana included the addition of a solar-powered light and fan.^6^

HCD, the process described by Kim et al., is an iterative approach to generating solutions that are firmly rooted in people, developing empathy with the end-user, generating an abundance of ideas, building tangible prototypes, and iteratively co-creating with people again. Four lenses of user desirability, business viability, technical feasibility, and sustainability typically serve as a framework, focusing on optimizing for the user experience while ensuring viability at scale. HCD has become a key element in the Center for Innovation and Impact^7^ at the United States Agency for International Development and at the Design for Health Initiative.^8^

The value of HCD for improving LLIN access and use is well recognized. However, the business viability of implementing HCD solutions that go beyond addressing only the technical feasibility is challenged by the current system. Currently, manufacturers are simply “vendors” rather than “partners,” with a focus on manufacturing uniform LLINs, with minimum specifications at the lowest possible price, in order to secure large tenders for mass distribution. With the sole focus on price, limited opportunities exist for innovations that address barriers to use, such as convenience, comfort, and personal lifestyle.^1^

Ultimately, our hope is that, as HCD emphasizes, people impacted by malaria will be enabled to guide us according to their priorities, how they live, and what is important to them. In turn, we will have a chance to create sustainable solutions that fit their lifestyles and will prevent malaria.

Our hope is that people impacted by malaria will be enabled to guide us according to their priorities, how they live, and what is important to them.

With the uncertainty of future global health funding,^9^ it is more important than ever that we optimize available resources to segment LLIN delivery strategies—that is, facilitate the growth of a consumer market for those who can afford to make purchases and concentrate public health resources on those who cannot, offering a diversity of LLIN products that fit the needs and preferences of each.

In his commentary on the survival of the Global Fund to Fight AIDS, Tuberculosis and Malaria, Richard Horton notes^10^:

*There are, of course, other questions the Fund must consider. How far should it embrace the private sector? What is its strategy for middle-income countries? How can the Fund leverage domestic investments …?*

In the context of LLIN distributions, this could be rephrased: How do we facilitate a commercial LLIN sector, what is the strategy for middle-income families, how can the Fund leverage consumer investments?

Kim et al.^1^ provide a step in that direction:

*We have since shared our consumer insights and preliminary ideas for new design features with current manufacturers globally who supply LLINs in Ghana. We hope these partners take this information into consideration as they make decisions about current and future LLIN supply, demand, and marketing and will pursue pilot testing of new net designs for the private sector retail market in Ghana.*

They conclude with a statement that seems to sometimes be lost on policy makers:

*For a health technology such as the LLIN to produce a benefit, it has to be used.*

There is much to be learned from consumer product development and HCD to solve complex problems for which we thought, with our hubris, we already knew the simple solution.
